# Fundus autofluorescence and optical coherence tomography of a macular cherry-red spot in a case report of sialidosis

**DOI:** 10.1186/s12886-016-0201-9

**Published:** 2016-03-22

**Authors:** Wenjun Zou, Xin Wang, Guohong Tian

**Affiliations:** Department of Ophthalmology, Nanjing Medical University Affiliated Wuxi Second Hospital, Wuxi, 214002 China; Department of Ophthalmology, Eye Ear Nose and Throat Hospital, Fudan University, 83 Fenyang Road, Shanghai, 200031 China

**Keywords:** Sialidosis, Cherry-red spot, Fundus autofluorescence, Optical coherence tomography

## Abstract

**Background:**

Sialidosis is a rare lysosomal storage disorder characterized by deficiency of alpha-N-acetyl neuraminidase. The macular cherry-red spot, which could be important for diagnosis, is a distinctive feature of its ocular manifestation. We evaluated the fundus autofluorescence (FAF) and optical coherence tomography (OCT) images of a juvenile patient who presented with vision decrease and was later confirmed with genetic sialidosis.

**Case presentation:**

A 13-year-old Chinese male presented with bilateral decreased vision over the past 2 years before his initial visit. Funduscopic examination revealed a macular cherry-red bilateral spot. FAF showed hyperreflective areas surrounding a central hyporeflective fovea in both eyes. OCT revealed increased reflectivity in the ganglion cell layer in both maculae without a definite boundary between the hyperreflective and normal areas. These findings suggested that lipofuscin had accumulated in the retinal ganglion cells, which is a distinctive ocular feature in metabolic central nervous system (CNS) disorders. He was later confirmed with genetic sialidosis.

**Conclusions:**

FAF and OCT images are very sensitive and useful techniques for diagnosing lysosomal storage disease of the CNS, and are helpful in evaluating the extent of damage in retinal ganglion cells.

## Background

The macular cherry-red spot was first named by Bernard Sachs. It is a very distinctive ocular manifestation of central retinal artery occlusion, traumatic retinal edema, and many neurological metabolic disorders such as Sandhoff disease, galactosialidosis, GM1 and GM2 gangliosidosis, and sialidosis types I and II [1]. Sialidosis is a rare lysosomal storage disorder characterized by deficiency of alpha-N-acetyl neuraminidase. It is transmitted as an autosomal recessive trait and is caused by a mutation in the neuraminidase (*NEU*) gene. There is a wide clinical spectrum ranging from nearly asymptomatic to severe presentations. The clinical features of sialidosis type I include late-onset, cerebellar ataxia, myoclonic epilepsy, nystagmus, as well as progressive visual loss [2–4]. The macular cherry-red spot is a very distinctive ocular feature for differential diagnosis of various CNS storage diseases [5]. We evaluated the fundus autofluorescence (FAF) and the optical coherence tomography (OCT) imaging in a juvenile patient presenting with a vision decrease, who was later confirmed with genetic sialidosis.

## Case presentation

**Fig. 1:**
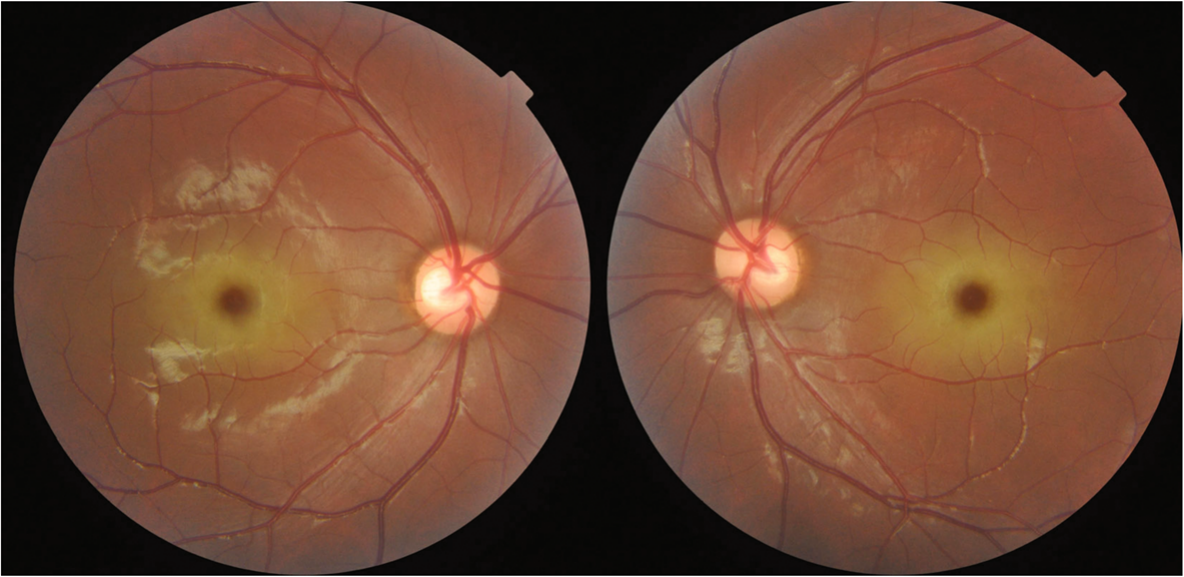
Fundus photographs, showing a cherry-red spot in both maculae

A 13-year-old Chinese male complained of vision decreased over the past 2 years, and had an unbalanced gait as reported by his parents. He was a high school student with normal intelligence and without smoking or alcohol abuse. His past medical history and family history were unremarkable. A neuro-ophthalmological examination revealed the patient to be alert and oriented. The best-corrected visual acuities (BCVA) were 20/100 OU. Color vision (Ishihara plates) was 1/8 OU. Slit lamp examination showed a punctate cataract and funduscopic examination revealed macular bilateral cherry-red spots and normal optic discs (Fig. 1). There was no nystagmus. The deep tendon reflexes were generally decreased with ataxia and slight myoclonus of the upper and lower limbs.

**Fig. 2:**
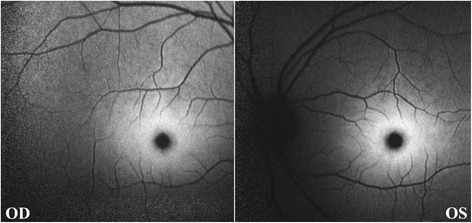
Fundus autofluorescence (FAF) imaging, showing bilateral hyperreflective areas surrounding the fovea

**Fig. 3:**
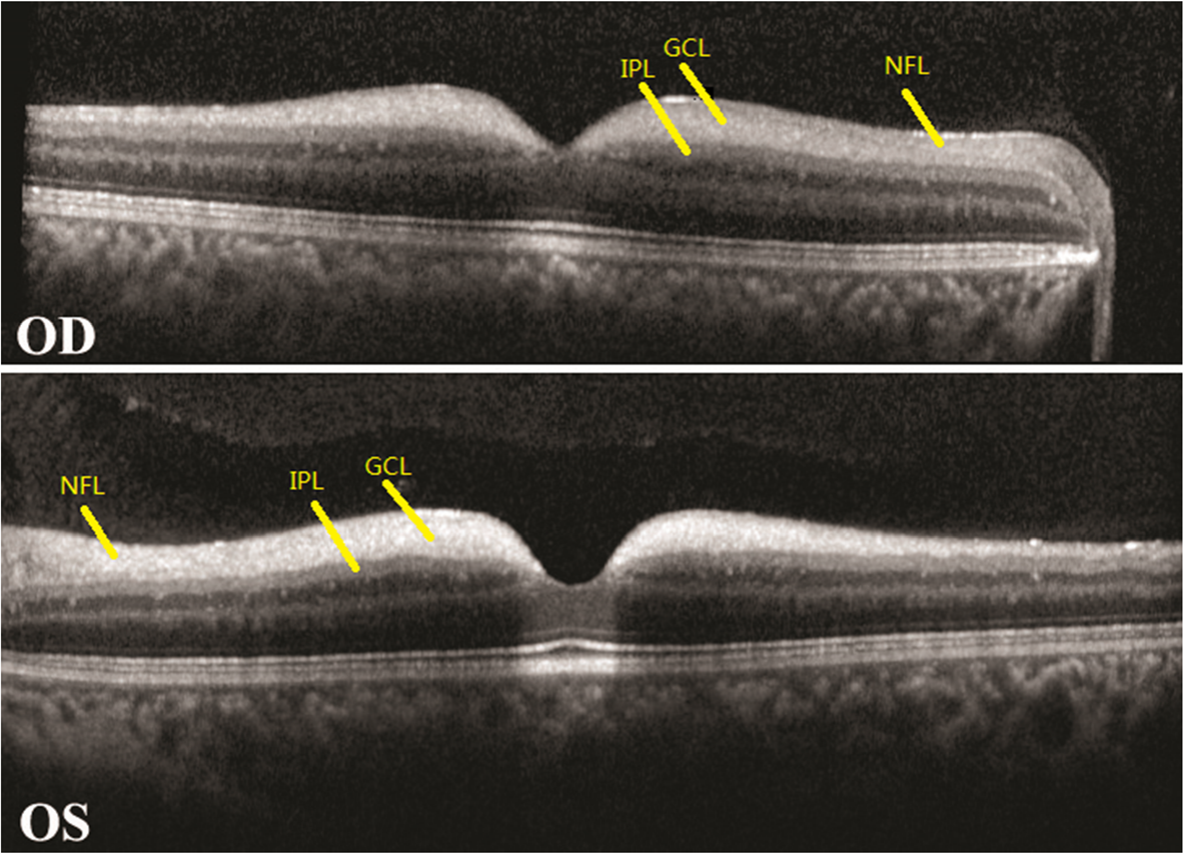
Optical coherence tomography (OCT) imaging, showing thickening and increased reflectivity in the ganglion cell layer (GCL) in bilateral eyes. The boundary between the GCL and the nerve fiber layer (NFL) is unclear. IPL = inner plexiform layer

FAF images (Heidelberg Engineering, Heidelberg, Germany) showed a patch of hyperreflective areas surrounding a hyporeflective fovea in both eyes (Fig. 2). OCT revealed increased reflectivity in the ganglion cell layer in both maculae, which corresponded to the hyperreflective areas on FAF; however, the boundaries between the hyperreflective and normal regions were not clear (Fig.3).

Laboratory test results showed normal levels of β-galactosidase, hexosaminidase A and B, arylsulfatase A, and β-galactocerebrosidase. Genetic analysis revealed two compound heterozygous mutations in the *NEU1* gene.

Because there was no treatment for sialidosis, the patient was only followed-up by his ophthalmologist and neurologist. His visual function was stable after a follow-up of 1 year.

## Conclusions

Sialidosis is a rare autosomal recessive disorder resulting from a deficiency of alpha-N-acetyl neuraminidase caused by a mutation in the *NEU gene* located on 6p21.33. This results in abnormal intracellular accumulation of sialyloligosaccharides, especially in brain neurons and in ganglion cells of the retina. A definitive diagnosis involves confirmation of a *NEU* gene mutation or a deficiency of neuraminidase activity in leukocytes and cultured fibroblasts using skin biopsies [2, 3].

Sialidosis is clinically classified as two major phenotypes: type I, a cherry-red spot myoclonus syndrome without dysmorphism, and type II, a more severe infantile form with dysmorphism [4]. Our case presented with bilateral cherry-red spots and myoclonus without mental deterioration and dysmorphism, which was consistent with type I.

The retinal ganglion cells are part of the CNS neurons. Retinal ganglion cell microstructure can be observed using an ophthalmoscope or by OCT. In the OCT images of the patient, abnormal hyperreflective areas were detected in the ganglion cell layer; however, the boundaries between the hyperreflective areas and the normal retinas were not clear [6, 7]. FAF imaging of the patient revealed bilateral hyperautofluorescent lesions surrounding a small hyporeflective center, the fovea. Because lipofuscin, which can be detected by excitation at 488 nm, is the primary source of retinal autofluorescence, the hyperreflective areas in retinal lesions indicate intracytoplasmic accumulation of lipofuscin-like granules from a metabolic disease such as sialidosis [8]. There are two interpretations for the retinal cherry-red spots in sialidosis. One is the deposition of lipofuscin granules in the ganglion cell layer in the perifoveal areas that result in a white patch around the fovea. Alternatively, the foveal pit lacks ganglion cells, and thus continues to maintain its reddish appearance [9].

In conclusion, when evaluating a patient with a cherry-red spot in the macula, which is not due to arterial occlusion or trauma, the diagnosis should consider various forms of lysosomal storage diseases. It is important to note that ophthalmic images using FAF and OCT are very sensitive and useful methods for detecting neurological metabolic disorders presenting with a cherry-red spot in the macula.

## Consent

Written informed consent was obtained from the parents (because the patient was under 16 years of age) for publication of this case and any accompanying images. A copy of the written consent is available for review by the editor of this journal.

## Abbreviations

BCVA: best corrected visual acuities
CNS: central nervous system
FAF: Fundus autofluorescence
GCL: ganglion cell layer
IPL: inner plexiform layer
NEU: neuraminidase
NFL: nerve fiber layer
OCT: optical coherence tomography
